# Assessment of knowledge, attitude and practice of mothers/caregivers on infant and young child feeding in Assosa Woreda, Assosa Zone, Benshangul Gumuz Region, Western Ethiopia: a cross-sectional study

**DOI:** 10.1186/s13690-021-00690-5

**Published:** 2021-09-26

**Authors:** Dawit Getachew Assefa, Tigist Tekle Woldesenbet, Wondowsen Molla, Eden Dagnachew Zeleke, Timsel Girma Simie

**Affiliations:** 1 College of Health Sciences, Center for Innovative Drug Development and Therapeutic Trials for Africa (CDT-Africa), Addis Ababa University, P.O. Box 9086, Addis Ababa, Ethiopia; 2 Department of Nursing, College of Health Science and Medicine, Dilla University, Dilla, Ethiopia; 3 Department of Public Health, School of Graduate Studies, Pharma College, Hawassa, Ethiopia; 4 Department of Midwifery, College of Health Science and Medicine, Dilla University, Dilla, Ethiopia; 5 Department of Midwifery, College of Health Science, Bule-Hora University, Bule-Hora, Ethiopia; 6 Department of Anesthesiology, College of Health Science and Medicine, Dilla University, Dilla, Ethiopia

**Keywords:** Infant and young child feeding, Knowledge, Attitude, Practice

## Abstract

**Background:**

Through the health extension package, Ethiopia had practiced infant and young child feeding. However, infant and young child feeding (IYCF) practice has been poor. Hence, in this study, the knowledge, attitude, and practice of the mothers/caregivers on infant and young child feeding were assessed.

**Methods:**

A cross-sectional study was carried out among 486 mothers/caregivers from Assosa Districts in the Assosa Zone of Benshangul Gumuz Region, Western Ethiopia. A semi-structured interviewer-administered questionnaire was used. To isolate independent predictors for good knowledge, good practice, and favorable attitude of the mothers/caregivers related to child feeding, multivariable logistic regression analyses were performed.

**Results:**

Out of 486 study participants, 456 (93.8 %) of mothers had good knowledge, 432 (88.9 %) had a positive attitude, and 380 (78.2 %) mothers had good practice of IYCF practice recommendations. Furthermore, age of mothers, educational status of the mother, place of delivery, father’s educational status, father’s involvement & support, previous knowledge about IYCF, discussion with their husband about IYCF, and ANC follows up were significantly associated with mother’s knowledge on IYCF recommendation.

**Conclusions:**

Overall mothers had good knowledge and a positive attitude about IYCF practices. To support IYCF practices, behavior change communications intervention strategies should be introduced in mothers to bridge the gap between knowledge and practices.

## Background

Infant and young child feeding (IYCF) consists of initiation of breastfeeding within 1 h of birth, exclusive breastfeeding (EBF) for 6 months, a continuation of breastfeeding for up to 2 years and beyond the introduction of complementary foods, minimum dietary diversity, minimum meal frequency, minimum acceptable diet, and consumption of iron-rich or iron-fortified foods [1–3]. IYCF has a major role in determining the nutritional status of children, maximizing the growth rate of a child at early years of life [2, 4], and has great potential for reducing under-five malnutrition and thereby affecting child mortality rate. Also, to improve the child health and development outcomes in poorly resourced communities, improved IYCF practices are crucial [5]. Hence, factors, such as the knowledge, attitude, and practice (KAP) of mothers/caregivers on infant and young child feeding in this critical time are very important for child health, growth, and development [4, 6–8].

During optimal complementary feeding (CF) program the quantity and quality of food, frequency, and timeliness of feeding, food hygiene, and feeding during or after illness are highly considered [9]. Besides these facts, inadequate complementary feeding practice of 6 months to 2 years old children is a major problem [4]. In low-income countries (LICs) improving the nutrition of infants and young children is a top priority for human development [5]. However, according to the 2020 world health organization’s (WHO) report, in low- and middle-income countries under-nutrition was linked to 45 % of deaths among children under 5 years of age. In the same year, 47 million children under 5 years of age are wasted, and 14.3 million are severely wasted and 144 million are stunted [3]. Inappropriate nutrition can also lead to childhood obesity which is an increasing public health problem in many countries [3].

Optimizing nutrition early in life including the 1000 days from conception to 24 months ensures the best possible start in life, with long-term benefits [10]. Especially, breastfeeding is one of the most effective ways to ensure child health and it could prevent 13 % of deaths occurring in children less than 5 years of age globally, while appropriate complementary feeding practices would result in an additional 6 % reduction in under-five mortality [11]. However, according to the current WHO report, nearly 2 out of 3 infants are not exclusively breastfed for the recommended 6 months a rate that has not improved in 2 decades [3].

In Sub-Saharan African regions, micronutrient deficiencies, poor quality of complementary foods, suboptimal infant feeding practices, and frequent infections have mainly contributed to the high mortality among infants and young children [12]. Since 2004 to improve feeding practice IYCF guideline was developed and implemented in Ethiopia. However, concerning all three IYCF practices (breastfeeding status, number of food groups, and times they were fed) the feeding practices of only 7 % of children aged 6 to 24 months meet the minimum standard [13], and infant and young child feeding (IYCF) practice has been poor [14, 15]. Poor child feeding practices, inadequate quantities, and inadequate quality of complementary foods have a severe consequence on health and growth in children less than 2 years of age [16]. Therefore, this study was aimed to assess the knowledge, attitude, and practice of the mothers/caregivers on infant and young child feeding in Assosa Woreda, Assosa Zone, Benshangul Gumuz Region (BGR), Western Ethiopia.

### Specific objective

To assess the maternal attitude towards the infant and young child feeding practices.

To assess the maternal practice regarding the infant and young child feeding practices.

To assess the maternal knowledge regarding the infant and child the feeding practices.

To identify the associated factors a of mother’s knowledge on IYCF.

## Methods

### Study setting and population

the community-based cross-sectional study design was conducted in Assosa district, Ethiopia from May 20 -June 30, 2020. Assosa District is one of the seven administrative districts of Assosa zone, BGR, Ethiopia. The district is located 20 km away from Assosa, the capital city of BGR, and685 kilometers from Addis Ababa, Western Ethiopia. There are a total of 18,828 under-five children and 6,773 under 2 years of age infants [17]. The study populations were all mothers/caregivers whose children were 0–24 months in selected districts. A multistage sampling method was employed. Nine sub-districts were selected from sub-district using a simple random sampling technique and from each sub-district participants were selected based on the proportion of children under the age of two in each kebele. Community Health Extension Workers (HEW) were provided the lists of all mothers who had a child under the age of two in each sub-district. A systematic random sampling method was employed to select study participants. When the household had more than one eligible child, one child was selected via a lottery method.

### Eligibility criteria

#### Inclusion criteria

➢ Mothers whose infant is under the age of two.
➢ Child families who were permanent residents in the study area.
➢ The mother agrees and gives her consent to the study or others criteria.

#### Exclusion criteria

➢ Children without their biological mother or actual caregivers.
➢ Mothers with infants who are seriously ill and unable to communicate from any cause.

### Sampling technique

A multistage sampling method was employed. Nine sub-districts were selected from 72 sub-districts using a simple random sampling technique and from each sub-district participants were selected based on the proportion of children under the age of two in each kebele. Community Health Extension Workers (HEW) were provided the lists of all mothers who had a child under the age of two in each sub-district. A systematic random sampling method was employed to select study participants. When the household had more than one eligible child, one child was selected via a lottery method.

### Sample size determination

The sample size was determined using the formula of sample size determination for single population proportion n=(*Za*/2)^2^ *P* (1 − *p*)/d^2^. By the following assumptions: The level of confidence (α) is taken to be 95 % (Z1-α/2 = 1.96), and the margin of error (d) is taken to be 5 % [0.05]. The proportion (p) of the prevalence of practice on mother knowledge in IYCF was 28.7 % (4). The calculated sample size was 495 mothers/caregivers.

### Study variables

#### Dependent variable

Mothers/caregiver knowledge, attitude, and practice on infant and young children feeding at the age of 0 to 24 months.

#### Independent variables

The independent variables were maternal socio-economic and demographic factors, obstetrics and medical factors, maternal care utilization ANC, and breastfeeding practice.

### Data collection and data quality assurance

Face to face interview was administered by a nurse after explaining the objectives when women were free and in a comfortable condition at their home or health facility. Data were collected using structured and pre-tested questioners. The questionnaires were first prepared in English, and translated into the local language (Amharic). The data collectors were given training on the process of data collection and during the data collection, consistent and accurate data were cheeked daily.

### Data management and analysis

After checking the completeness and appropriateness, the data was coded and entered Epi Data 3.2, check for missing values and outliers, and was exported to SPSS 25 for data analysis. Descriptive frequencies and percentages were used to present the study results. First, a descriptive statistical analysis will be used, and mean, standard deviation (SD) was used to describe the socio-demographic characteristics and prevalence of knowledge, attitude, and practice on child feeding. Bivariate analysis was employed to identify the candidate variables for multivariable analysis at *p* < 0.025 in binary analysis. Moreover, the proportion difference between the KAP by the socio-demographic background was analyzed by using Pearson’s Chi-square tests after checking the assumptions. The multivariable results are reported as adjusted odds ratio (AOR) with 95 % CI. The significance of the results was declared at *p* < 0.05.

### Ethical consideration

This proposal was submitted to Pharma College, school of graduate public health to be approved and obtaining a letter of clearance. An official letter of cooperation was also be given to the Assosa district health office and the Assosa zone health office. The Assosa district health office was asked for an official letter to get permission. Data collectors were trained how to handle confidentiality and privacy using the consent form attached to each questionnaire. Confidentiality was assured by excluding their name during the period of data collection.

## Results

### Socio-demographic characteristics of the respondents

**Fig. 1:**
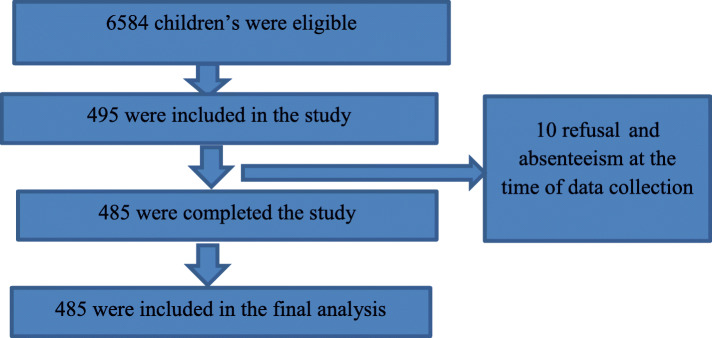
Study flow diagram

The total response rate of the study was 486 (98.2 %). The reason for non-response was due to refusal and absenteeism at the time of data collection (Fig. 1).

The mean (± SD) age of the participants was 29.5(5.4) years. The majorities were illiterate of which 232 (47.7 %) were unable to read and write, 412 (84.8 %) of them lives in rural areas, and 248 (51.0 %) of them were housewives (Table 1).

**Table 1:** Maternal and child socio-demographic data in Assosa Zone, BGR, Western Ethiopia, 2020

Variables	Frequency	Percent (%)
**Age of mother**		
< 24	82	16.9 %
25–29	178	36.6 %
≥ 30	226	46.5 %
**Age of children (years)**		
0-6 months	156	32.1 %
6–12 months	172	35.4 %
12–18 months	86	17.7 %
19–24 months	72	14.8 %
**Sex**		
Male	294	60.5 %
Female	192	39.5 %
**Place of residence**		
Rural	412	84.8 %
Semi-urban	74	15.2 %
**Family size**		
< 3	130	26.7 %
4–6	260	53.5 %
> 6	96	19.8 %
**Education status of the mother**		
Unable to read and write	232	47.7 %
Able to read and write	108	22.2 %
Primary education	94	19.3 %
Secondary education	32	6.6 %
College and above	20	4.1 %
**Education status of the father**		
Unable to read and write	188	38.7 %
Able to read and write	120	24.7 %
Primary education	84	17.3 %
Secondary education	54	11.1 %
College and above	40	8.2 %
**Occupational status of the mother**		
Housewife	248	51 %
Farmer	192	39.5 %
Merchant	30	6.2 %
Daily laborer	4	0.8 %
Gov’t employee	12	2.5 %
**Parity**		
1	416	85.6 %
2	68	14.0 %
≥ 3	2	0.4 %

### Knowledge of respondents about IYCF practices

Overall, 456 (93.8 %) mothers had good knowledge of IYCF practice recommendations. The majority, 408 (84 %) of participants realized that breast milk was the first feed that should be consumed by the child after birth within a 1 h of birth, and 416(85.6 %) women knew that exclusive breast-feeding means that an infant should receive only breast milk up to 6 months of life. Four hundred four (83.1 %) respondents identified the exact time of complementary feeding initiation. Among all respondents, the majority 442(90.9 %) knew mothers should take healthy food. Furthermore, four hundred forty-six (91.8 %) of respondents knew that breastfeeding could strengthen the bond between mother and child. More than three quarter (76.1 %) of respondents knew that breastfeeding helps to child and the majority 412(84.8 %) of respondents knew that frequent breastfeeding is required when a child is sick (Table 2).

**Table 2:** Knowledge of mother towards IYCF practices

Variable	Frequency	Percent (%)	
**Colostrum is important for baby**			
Yes	394		81.1 %
No	44		9.1 %
Don’t know	48		9.9 %
**A neonate should start breastfeeding within 1 h of birth**			
Yes	408		84 %
No	42		8.6 %
Don’t know	36		7.4 %
**An infant should exclusively breastfeed for the first 6 months**			
Yes	416		85.6 %
No	56		11.5 %
Don’t know	14		2.9 %
**An infant should start complementary food at 6 months**			
Yes	404		83.1 %
No	64		13.2 %
Don’t know	18		3.7 %
**Lactating mothers should take healthy food**			
Yes	442		90.9 %
No	6		1.2 %
Don’t know	38		7.8 %
**Did you wash your breast before breastfeeding**			
Yes	248		51.0 %
No	238		49.0 %
**A snack should give to the child**			
Yes	394		81.1 %
No	64		13.2 %
Don’t know	28		5.8 %
**BF can strengthen the bond between mother and child**			
Yes	446		91.8 %
No	36		7.4 %
Don’t know	4		0.8 %
**BF can prevent disease**			
Yes	226		54.7 %
No	162		33.3 %
Don’t know	58		11.9 %
**BF helps to child**			
Yes	370		76.1 %
No	96		19.8 %
Don’t know	20		4.1 %
**BF is important economically**			
Yes	252		51.9 %
No	190		39.1 %
Don’t know	44		9.1 %
**BF more frequently when a child is sick**			
Yes	412		84.8 %
No	74		15.2 %
Don’t know	-		
**Who is usually feed a child**			
Mother	436		89.7 %
Father	4		0.8 %
Sibling	44		9.1 %
Guardian	2		0.4 %
**Involvement and support of father on IYCF**			
Yes	398		81.9 %
No	60		12.3 %
Don’t know	28		5.8 %
**Did you discuss with husband about child nutrition and feeding**			
Yes	268		55.1 %
No	218		44.9 %
**Overall knowledge of IYCF**			
Good	456		93.8 %
Poor	30		6.2 %
**Source of information**			
TV/ Radio	12		2.5
Health facility	270		55.6 %
On ANC/PNC follow up	200		41.2 %
Relative/ Neighbor	4		0.8 %
Social media (Facebook, …)	-		
other	-		

*ANC* Antenatal care, *BF* Breastfeeding, *hrs* hours, *IYCF* Infant and young children feeding, *PNC* Post natal care, *TV* Television

### The attitude of respondents towards IYCF practices

Concerning attitude about IYCF, 410(84.4 %) participants agreed that breastfeeding should start immediately after delivery and 372(76.5 %) also agreed that exclusive breastfeeding for the first 6 months is necessary. Approximately three quarter reported that babies shouldn’t be given anything except breastfeeding until 6 months. Majorities 444(91.4 %) were agreed that complementary feeding should be started after 6 months (Table 3). Concerning the level of attitudes, more than half (88.9 %) of the participants had a positive attitude towards IYCF recommendations.

**Table 3:** Attitude of Respondents towards IYCF Practices

Attitude question	Disagree	Not sure	Agree
**Breastfeeding should start immediately after delivery**	44(9.1 %)	32(6.6 %)	410(84.4 %)
**Babies shouldn’t be given anything except BF ≤ 6 months**	88(18.1 %)	26(5.3 %)	372(76.5 %)
**A child can be given butter, sugar, and water ≤ 6 months**	276(56.8 %)	22(4.5 %)	188(38.7 %)
**Complementary feeding should be started after 6 months**	36(7.4 %)	6(1.2 %)	444(91.4 %)
**A formal meal is more convenient**	322(66.2 %)	28(17.3 %)	136(28 %)
**Cow milk is more convenient**	370(76.2 %)	8(1.6 %)	108(22.2 %)
**BF should continue up to 2 years**	22(4.5 %)	2(0.4 %)	462(95 %)
**A child should be breastfeeding 10 and more than 10/24 hrs**	18(3.7 %)	4(0.8 %)	464(95.5 %)
**The child food to eat at one time should include VitA, and Fruit, etc…**	34(6.9 %)	14(2.9 %)	438(90.1 %)
**A snack should be given to the children between meal**	12(2.4 %)	30(0.8 %)	444(91.3 %)
**Serving balanced foods prevent malnutrition disposal**	28(5.7 %)	16(3.3 %)	442(90.9 %)
**Serving only starchy food prevent malnutrition**	274(56.4 %)	38(7.8 %)	174(35.8 %)
**Malnutrition can be caused by disease**	76(15.6 %)	28(5.8 %)	382(78.6 %)
**Serving indigenous fruit/vegetable can keep children healthy**	106(21.8 %)	24(4.9 %)	356(73.2 %)

*BF* Breastfeeding, *hrs* hours

### IYCF practices

A total of 406(83.5 %) children were breastfed within an hour of their birth and the majority 370(76.1 %) of children had more than ten frequencies of breastfeeding. In addition, three hundred eight (87.2 %) of children were exclusively breastfed for the first 6 months of life and nearly half 240(49.4 %) children started with complementary feeds at 6 months. However, 334(68.7 %) children were complimentary food at least 3times/day. Overall, 380 (78.2 %) mothers had a good practice on IYCF (Table 4).

**Table 4:** IYCF practice among respondent

Variables	Frequency	Percent (%)
**At what time you started BF after birth**		
Within 1 h	406	83.5 %
After 1 h	80	16.5 %
**Frequency of BF in the last 24 h**		
< 10 times	116	23.9 %
≥ 10 times	370	76.1 %
**Exclusive breastfeeding for the first 6 months**		
Yes	308	63.4 %
No	178	36.6 %
**The time you started complementarily**		
< 6 months	172	35.4 %
At 6 months	240	49.4 %
> 6 months	74	15.2 %
**For how many years continued BF**		
< 2 years	88	18.1 %
≥ 2 years	398	81.9 %
**Minimum meal frequency of complementary food**		
Once	30	6.2 %
Twice	122	25.1 %
≥three times	334	68.7 %
**Overall status of IYCF practice**		
Good	380	78.2 %
Poor	106	21.8 %

*BF* Breastfeeding, *IYCF* Infant and young children feeding

### Factors associated with mothers’ knowledge

In bivariate analysis, the data showed that there was no association between mothers’ knowledge and the variables analyzed, these variables include the place of the respondent, sex, marital status, religion, occupation, family size, etc.

In the binary logistic regression analysis age of mothers, educational status of the mother, place of delivery, educational status of the father’s, father’s involvement & support, previous knowledge about IYCF, discussion with their husband about IYCF and ANC follow up were statistically associated with mothers knowledge on IYCF recommendation (Table 5).

**Table 5:** Factors associated with mother’s knowledge regarding IYCF

Variables	Mothers’ knowledge of IYCF		COR(95 %CI)	AOR(95 %CI)	*P*.value
	**Poor**	**Good**			
**Age of mothers**					
< 24 years	8	74	0.8(0.33,1.92)	0.29(0.1,0.85)*	0.024
24–29 years	4	174	3.76(1.25,11.3)	1.92(0.58,6.34)	
≥ 30 years	18	208	1	1	
**Educational status of the mother**					
Literate	24	316	1.77(0.71,4.43)		
Illiterate	6	140	1		
**Place of delivery**					
Home	8	32	1	1	0.009
Health institution	22	424	4.82(1.99,11.68)	4.47(1.46,13.68)	
**Father educational status**					
Illiterate	22	286	1		
Literate	8	170	1.64(0.71,3.75)		
**Father involvement &support**					
Yes	22	376	1.71(0.74,3.98)		
No	8	80	1		
**Did you ever heard information about IYCF**					
Yes	24	436	5.45(2.0,14.8)	3.66(1.16,11.61)	0.027
No	6	20	1	1	
**Did you discuss with your husband about IYCF**					
Yes	10	256	2.56(1.17,5.59)		
No	20	200	1		
**ANC follow up**					
Yes	2	278	21.9(5.14,92.9)	12(4.84,25.1)	0.001
No	28	178	1		

*ANC *Antenatal care, *AOR* Adjusted odd ratio, *CIs* Confidence intervals, *COR* Crude odds ratio, *IYCF* Infant and young children feeding

After controlling the effect of other variables (confounders), the likelihood of a good knowledgeable mother was 71 % times less likely for mothers age between < 24 years old than mothers who were ≥ 30 years old. Additionally, mother who had delivered in health institution were 4.47 time more knowledgeable than who had delivered at home.

More mothers who had ever heard information about IYCF were 3.66 times more knowledgeable than had not ever heard information about IYCF. Furthermore, mothers who had ANC follow-up were 12 times more knowledgeable than their counterparts who had no ANC follow-up.

## Discussion

To our knowledge, this study was the first to be conducted in Assosa Zone, BGR, and Western Ethiopia. It was conducted to assess Knowledge, attitude, and practice towards IYCF, and associated factors among mothers’ on IYCF.

Overall, 93.8 % of mothers had good knowledge of IYCF practice recommendations. Mothers who have good knowledge of IYCF recommendations were more likely to have better feeding practices than mothers who have poor knowledge [18, 19]. The finding that we get from this study was higher than the study findings in Bennatsemay woreda (45.7 %), and Nairobi city (49.5 %) [20, 21]. On the other hand, this finding was lower than the study findings in Shebele Zone, in India, and Solapur city [22–25]. This disparity may be explained by the fact that most of the mothers in this study had no formal education on infant feeding [25], the time gap between studies, and the difference in the study settings; since the current study was done among mothers in with lower socio-economic status whereas the former studies included mothers in the Woreda with better socio-economic characteristics.

The mother’s general knowledge base was higher evidenced than an attitude about IYCF practices. Recent studies reported that the IYCF attitude of participants was limited [5, 14]. On the contrary, other studies found a desirable attitude of mothers and fathers towards IYCF practices. However, even though the mothers perceived good knowledge, one-fourth of fathers influenced the earlier stopping of breastfeeding. This is due to a common belief in Papua New Guinea that whilst a woman is breastfeeding a couple should not resume sexual relations. To enhance good IYCF practices, the mothers/caregivers need to have both good knowledge and attitude towards IYCF [23]. The current study was also supported a similar finding.

For the poor growth of children, poor practices can be one of the reasons. In a study conducted in West Bengal, India IYCF practice was higher and it was significantly related to the age and educational status of the mothers. One such study in Kerala reported that 84.1 % of mothers exclusively breastfed their children [26]. It was reported that despite mothers having good knowledge about IYC, mothers’ practice in feeding the child was poor [22–24]. Hence, despite their knowledge about IYCF hands-on training and practical exposure is the key to improve feeding practices.

It has been reported that 63.4 % of mothers breastfed their child for the first 6 months, 65 % of children were introduced to complementary feeds after the 6 months. Breast milk is the ideal food for infants. It is safe, clean, and contains antibodies that help protect against many common childhood illnesses [3, 16]. In this study majority of mothers initiated breastfeeding the child within 1 h of birth which is higher than the previous study [27]. One former study was reported that the recommended duration for early breastfeeding recognized by 92 % of the mothers, 96.9 % knew about the duration for exclusive breastfeeding, although only 25 % knew about the time to start complementary feeds [22]. In other previous studies, it was reported that 65.8 % of infants were not initiated breastfeeding within 1 h of birth [2], 17 % were breastfed exclusively [25], 74 % were breastfed for 12 months and only 41 % were initiated with complementary feeds at age of 6 months [28].

This study shows that the likelihood of a good knowledgeable mother was 71 % times less likely for mothers age between < 24 years old than mothers who were ≥ 30 years old. A similar result was reported from a study finding in Nairobi city, Kenya [21]. Additionally, mother who had delivered in health institution were 4.47 time more knowledgeable than who had delivered at home.

More mothers who had ever heard information about IYCF were 3.66 times more knowledgeable than had not ever heard information about IYCF. Furthermore, mothers who had ANC follow up were 12 times more knowledgeable than their counterparts who had no ANC follow-up. This finding is in agreement with the study finding in northern Ethiopia and Arba Minch Zuria [29, 30]. Mothers who had ANC follow-up were more likely to be counseled by professionals on IYCF, which have a direct contribution to improve their knowledge level [31].

The study recommended revitalizing and expanding the baby-friendly hospital initiative and establishing breastfeeding intervention programs for protection, promotion, and support of breastfeeding. We also recommend for health workers to provide information on the involvement of the male partner in antenatal care is integrated into the public health system and education on infant and young child feeding recommendations should be strengthened during antenatal care visits and using mass media especially for mothers with lower educational status to fill up of this gap.

## Conclusions

Overall, the mothers had good knowledge and a fair attitude about IYCF practices. Age of mothers, place of delivery, previous information about IYCF, and ANC follow-up were statistically associated with mother’s knowledge of IYCF recommendations. Behavior change communications intervention strategies, which would support IYCF practices, should be introduced in mothers to bridge the gap between knowledge and practices.

### Strength and limitation of the study

The questionnaire utilized for this study is based on the WHO IYCF Indicators parameters. This study was the first to be conducted in Asossa. However, the limitation of the study is that it was conducted among lactating mothers that opted for post-natal services, and hence, the findings of this study may not be representative of the situation of infant and young child feeding practices for the community at large.

## Data Availability

Data will be available upon request from the corresponding authors.

## Abbreviations

ANC: Antenatal Care
AOR: Adjusted Odd Ratio
BF: Breast Feeding
BGR: Benishangul Gumuz Region
CF: Complimentary feeding
CIs: Confidence Intervals
CSA: Central Statistical Authority
COR: Crude Odds Ratio
EBF: Exclusive Breast Feeding
EDHS: Ethiopian Demographic and Health Survey
hrs: Hour
HHs: Household
IHRERC: Institutional Health Research Ethical Review Committee
IYCF: Infant and Young Children Feeding
KAP: Knowledge, Attitude and Practice
LICs: Low- income countries
OR: Odds Ratio
PNC: Postnatal Care
SD: Standard Deviation
SPSS: Statistical Program for Social Science
TV: Television
UNICEF: United Nation Children’s Fund
WHO: World Health Organization
